# “[Culture] Makes Each Country Unique, It’s Kind of like a Trademark.” Empirical Results on Students’ Perceptions of Culture and Space as Learning Prerequisite for Geography Lessons

**DOI:** 10.3390/ejihpe12020009

**Published:** 2022-01-25

**Authors:** Ronja Ege, Alexandra Budke

**Affiliations:** 1 Department VI—Spatial and Environmental Sciences, University of Trier, Cultural and Regional Geography, 54286 Trier, Germany; 2 Institute for Geography Education, University of Cologne, Gronewaldstraße 2, 50931 Cologne, Germany; alexandra.budke@uni-koeln.de

**Keywords:** cultural-geographic education research, culture and space, students’ ideas, transculturalality, imagination research, prevention of racism, questionnaire survey

## Abstract

Students’ everyday perceptions of culture and space have a significant impact on their social coexistence and should, therefore, be considered in geography lessons. The *other* or *foreign* is often negatively assessed. This perception is based on an essentialist understanding of cultural space but is inappropriate for a culturally diverse world. The concept of transculturality by Wolfgang Welsch offers a constructivist perspective on culture and space, which takes cultural globalization into account and avoids a stereotyped division of cultures. To prevent xenophobia, it is important to understand the everyday ideas about culture and space younger generations possess and the extent to which transculturality is integrated. The learning requirements of students provide a basis on which geography lessons can be developed that incorporate these concepts to counteract xenophobia. To determine everyday perceptions, 197 German 9th-grade students were asked. The selection of the sample was based on a declaration of consent from the parents and was not fully probabilistic. In addition to a preliminary study, 98 female and 99 male students were surveyed in a written questionnaire. The data analysis was realized in a mixed-method design, with qualitative content analysis and supplementary quantifications. The results of which suggested that culture and space are predominantly understood as nationally specific. Consequently, a transcultural understanding should be incorporated long-term into geography lessons.

## 1. Introduction

The violent death of African-American George Floyd in 2020 led to the revival of the anti-racism movement “Black Lives Matter.” The subsequent protests were directed against structural racism, a problem not only in the USA but globally. Mechanisms of distinction, separation, and hierarchization on a social and political level are central components of cultural racism. Cultural racism is based upon racially-motivated power differences, where certain groups of people are considered to be socio-culturally inferior due to their supposedly naturally given cultural characteristics such as religion or skin color [1]. Such a cultural orientation system builds upon the concept of *us and the others*, which believes that cultures should be homogeneous and cohesive and is thus essentialistic [2,3]. Cultural racism is not only found in migration policy. Current geography textbooks also contain examples of topics based upon essentialist concepts of culture, which are still taught [4].

Culture and space are omnipresent in geography textbooks across a wide variety of topics that can be related to migration and urban geography, among others. Additionally, specific regional examples of cultural concepts and space are incorporated into such texts, such as the role of women in India. Consequently, sensitization of the risks of commonly accepted understanding of culture and space present in such books should be of concern in geography education.

Among others, Alexandra Budke [5] showed, through an analysis of German geography textbooks, how binary differentiation mechanisms between cultures are promoted within geography education. For example, cultural differences are attributed to the natural connection between people and space (“indigenous people in the rainforest”) or religious affiliation [5] (p. 157). Stereotypes are formed through such assumed associations, which lead to culturalization and neglect of social diversity [6,7,8]. In addition, so-called major civilizations are reproduced, as the localization of *the orient* in atlases impressively illustrates [9]. The concept of major civilizations was promoted by the Geographers Albert Kolb [10] and Jürgen Newig [11], with the effect that space and culture conveyed as “naturally given”. Such positioning of culturally homogeneous groups in a geographical container space is dangerous, as nations or states are produced through geopolitical and legal interventions alone. However, such division can serve as a foundation for racism when such nations and states are linked to supposedly uniform ethno-cultural group [12]. The identification with a national culture occurs through the collective sharing of values, language, or behavior. Simultaneously, distinctions to other groups or nations are made thus that one’s own collective can be defined as *unique*. The valuation of one’s own collective is always more positive than that of other collectives in order not to undermine one’s own identity [3]. This positive self-portrayal of one’s own, supposedly ethnoculturally homogeneous group and its seemingly naturally-given, national location can lead to discrimination against people who are considered to have a different culture. The conception of identity here is based on the attribution of oneself. Both matching and differing characteristics mark whether or how individuals are integrated into a group. Only through the agreement as well as the difference can identification be accomplished, and identity exists. Belongings to groups on which identity is built are flexible and can change over time [3].

Currently, the concept of major civilizations is a recommended topic in the latest edition of the German core curriculum [13]. However, the danger of promoting xenophobia in society through teaching this topic is not sufficiently recognized. Additionally, other current educational concepts such as intercultural and global learning in geography education do not address the problem at all or sufficiently [14,15,16]. One approach that can help to prevent the aforementioned risks is the concept of transculturality by Wolfgang Welsch [17]. Its focus lies on commonalities and interconnections between cultures and considers ubiquitous globalization, which understands culture as a dynamic, human-made product [18]. As such, the reproduction of closed individual cultures is disregarded.

Overall, it is evident that there is both a demand for research on educational concepts in which the problems and deficits discussed are considered further, as well as a need to investigate how students think about culture and space. Consequently, geography lessons should be planned, thus they offer an alternative understanding of culture and space and thus help to reduce the aforementioned risks [14]. The following research questions have been formed to address this:

- Which everyday ideas about culture do the students possess?
- Which spatial references do students produce in the context of culture?

The first question serves to reveal the students’ basic understanding of culture. Forms and conditions in which culture exists for the students, such as the genesis of culture or which meanings it has for them, are determined. Question b. looks to consider the role spatial dimensions of culture play in students’ perceptions, with spatial references illustrated by using levels of scale. By explicitly addressing the question of place and space, a geographical perspective is taken into account [19].

In the following sections, theoretical approaches to the concepts of culture in geography and geography education are outlined. Subsequently, the research design and methodology outline the process of collection and analysis of data, which leads to the results of the study. A mixed-method design was employed, with both qualitatively open and quantitatively closed questions. The resulting questionnaire was completed by 250 German 9th-grade students and evaluated both using a content-analytical and descriptive-statistical approach. In conclusion, the significance of the results for further research, geography teaching, and geography education are discussed.

## 2. Main Concepts of Cultural Understanding

This chapter focuses on the different meanings of cultural concepts in society, sciences, geography, and geography education.

The diversity of the term culture and its application in such areas as media, everyday life, and sciences are highly segregated. This variation can be attributed to the changing objects of reference of culture, which are influenced and shaped by social developments. These developments are exemplified by the topics discussed at the annual Popular Culture Association/American Culture Association conference 2021. Experts discussed topics such as fitness culture, beer culture, children’s and young adults’ literature, and culture or comedy and humor. The variety of topics that were considered to be culturally-focused or as aspects of culture is also reflected in the well-known iceberg model by Hall [20]. Here, a distinction is made between clearly visible parts of culture (e.g., food, clothing, language) and invisible aspects that remain hidden, i.e., *under water* (e.g., values, feelings, communication styles), which influence the expression of the visible. Fundamentally, the various cultural terms can be distinguished through essentialist or constructivist understanding [5,21]. Essentialist understanding applies culture in the form of behavior or language as a spatial characteristic of belonging [22]. It is not only in everyday life that culture or cultural characteristics are equated with geopolitical space. Scientific disciplines such as intercultural psychology or pedagogy also make use of essentialist understanding as well as its application and impacts, side effects, interactions between cultures or through cultures, and create guides on how to handle foreign cultures [23,24,25]. An important component of the essentialist understanding is localizations of culture, which make a distinction between self-culture and foreign culture, especially in terms of national space, and, therefore, acknowledges orientation and identity (overview at [26]).

In contrast, constructivist understanding considers culture as a socially constructed phenomenon that has become part of social reality only through its recurring application in human practices of action [27]. The socially constructed contents of culture, which, for example, distinguishes a “German” from an “Italian” also function as political and social regulators and structures for life and one’s own perception [28,29] (p. 78). According to constructivist understanding, culture and cultural spaces are human-made and, therefore, do not “really” exist. Apart from the interpretation in terms of scientific theory, many concepts of culture primarily contribute to establishing meaning by providing comparisons on the basis of (cultural) differences and thus guarantee order [30].

### 2.1. Cultural Concepts in Geography and Geography Education

Scientific approaches on how culture can be understood also occur in geography and geography education. Regional studies, which are the historical core of geography, focused on the spatial classification of the earth. Auxiliary tools were used to determine natural demarcation criteria that were used to represent so-called “earth specificities” [31] (p. 4), which were described in detail by the geographer and educator Carl Ritter in his 1807 publication [32]. During an initial development in cultural considerations at the end of the 19th century, human relations in space were included as a component of geography, and the idea of the causal connection between nature (nation) and culture (people) developed into a central idea [33]. The philosophical approaches of Friedrich Hegel assumed that every nation carried a natural type of locality within it [34], which was also supported by the universal scholar and philosopher Gottfried Herder and emerged between and 1784 and 1791 [17], was very influential. He described cultures as enclosed, unique entities and equated them with the spatial expansion of an entire society [35]. Thus, the foundation for the concept of the cultural nation was set. In German geography, the same concept was predominantly established by the development of the *10 cultural spheres* by Karl Sapper [36], and later through the development of the concept of major civilizations by Albert Kolb [10], who divided cultures into trans-territorial, subcontinental conurbations. In some German federal states, these concepts remain an integral part of educational geography [8] as educational goals are set by the individual federal states.

New Cultural Geography was developed in the 1980s in the Anglo-Saxon region, triggered by the questioning of established and traditionally oriented categories of cultural spaces. Through further cultural developments within humanities (e.g., spatial and semiotic developments), antiquated approaches were replaced with the new paradigm of constructivism [35,37,38]. As a result, the research orientation of human and cultural geography changed, with culture understood to be a collective construct that reproduces itself through everyday actions that conceptualize space, i.e., loads it with meanings [34].

This anti-essentialist shift in perspective is also emerging in geography education. Instead of teaching rigid category systems, there is a shift to students being made aware of a diverse range of perspectives and for promoting a critical, questioning view, and concepts and ideas are not simply taken for granted [39]. Due to the omnipresence of culture and space in geography textbooks and thus most likely in the classroom, one goal should be to make students understand these concepts as socially produced negotiation processes [40]. Another way to question the simplified, stereotypical ideas and representations of culture and space with students is by using Welsch’s [17] concept of transculturality, which can demonstrate “cultural mixtures, similarities, overlaps and cultural change” to students [5] (pp. 159–160) and draw their awareness towards an alternative concept of culture and space.

### 2.2. Transculturality

“[The] ‘reality’ of culture [is] always also a result of our concepts of culture” [41] (p. 56). In his quotation, Wolfgang Welsch refers to the different ways (essentialistic and constructivist) in which culture can be understood and thus shape our views on social reality. He draws attention to the fact that there can also be a cultural reality that differs from the widespread essentialistic one developed by philosophers in the early 1990s. In his opinion, the essentialist sphere model founded by Gottfried Herder, in which cultures exist as closed, ethnically homogeneous units and are incapable of interacting with each other, does not correspond to contemporary cultural relations shaped by globalization [42]. Rather, cultures are based on similarities, transgressions, and interconnections. Wolfgang Welsch justified this by highlighting the diversity of lifestyles and forms of life that can be found in society but exist parallel to one another (e.g., “working-class settlement, alternative scene, villa district, heterosexual or homosexual orientation,” etc.) [42] (p. 13). These categories result in different cultural patterns that can be found worldwide and lead to cultural differentiation within (national) societies, states, regions, cities, and continents. The mutual influence and hybridization of cultures are driven by migration movements, as well as economic and technological developments. For example, the debates around women’s rights, human rights, or ecological awareness are occurring globally [42]. To Wolfgang Welsch, individuals are also considered transcultural since the identity of a person consists of the connection between the multiple cultural components he or she is confronted with in everyday life [16]. The philosopher refers to individuals who have “multiple cultural connections” and thus are “shaped by different countries of reference” as “cultural hybrids” [16] (p. 43) and [43] (p. 198).

Implementation of this new transcultural concept in the field of education is in its infancy. In the context of intercultural pedagogy, its use is often only discussed theoretically, and there is a lack of concrete applications [44,45,46]. It is only in didactics of foreign languages that transcultural learning is included as a concrete educational concept [47]. However, within geography education, it is non-existent. In order to counteract the aforementioned problems and dangers, such as culturalizations, stereotyping, and ethnocentrism, that teaching an essentialist understanding of culture and space causes, the implementation of transcultural learning in geography education would be considered to be useful. It is, therefore, necessary to first investigate whether students already possess a transcultural understanding and how it is composed. On this basis, in a further step, the prerequisites for an efficient transculturally-oriented geography lessons can be determined.

To reveal their transcultural pre-understanding, a survey of German students was conducted on the topic of culture and space. How the respondents’ ideas were collected and the results are presented subsequently.

## 3. Research Design and Methodology

The 2 major focuses of the investigation were culture and space, as well as consideration of subjective ideas around these, which were highly constructivist in character. To be able to externalize and reconstruct both aspects appropriately, a qualitative-open survey approach was used initially, which ensured that respondents’ conceptions were considered comprehensively. In addition, quantitative-closed methods were used to explore the complexity of the topics and to gain a condensed insight into the students’ perspectives and attitudes [48] and were used to estimate the quantities of certain attitudes and perspectives within the group of participants. The broad coverage of students’ conceptions and their generalization through quantitative closed methods led to the use of a mixed-methods design in both the survey and analysis (see Table 1). This also made it possible to relate the 2 data sets to each other and thus increase the degree of accuracy [49].

The core survey was a written questionnaire with open and closed questions, which was completed by a total of 197 German 9th-grade students at 2 gymnasiums and 1 comprehensive school. A preliminary exploratory study consisting of open questioning, which was completed by 53 German 9th-grade students from another gymnasium, was undertaken prior to the main data collection, which served to obtain an initial assessment of the range of ideas on the topic and to help design the closed questions of the core survey. The written survey was conducted during official class time and lasted about 30 min. During this time, the students were encouraged not to talk to each other. Dependent on the research question, it was possible to include questions in the pre-survey in the main survey as well, as no quantitative closed-ended questions were posed in the exploratory pre-study. Due to this, the total number (n) varies in the figures of the result.

Table 1 provides information on the procedure of the investigation, the main survey questions of the sub-studies, their research interest, and the method of data analysis.

The guiding idea of the explorative pre-study was to discover what ideas about culture and space the 9th-grade students possessed initially. The text material collected was systematically analyzed using content analysis according to Philipp Mayring [50,51]. Although the data were summarized to its essential components, the goal was to represent the spectrum of answers as broadly as possible. The results provided the basis for the creation of the core survey, particularly the closed questions (see Table 1).

The evaluation of the main survey was also carried out by means of a text-structuring content analysis and by using ordinal scaling analysis steps. Thus, qualitative and quantitative analysis steps were used [51,52]. A category system was developed by combining an inductive-deductive approach for the content-structuring analysis [52], which made the coding of the data material possible. Table 2 shows an excerpt of the developed category system used for the content analysis and provides detailed information in the form of subcategories, explanations, and text examples (referring to Part A of the core survey in Table 1).

Written responses by the participants were categorized according to the categories in Table 2 and analyzed by using content analysis. The process revealed structures and patterns contained within the text material [14]. The category *transcultural approaches* were created in order to reveal the initial understanding of transcultural by the students (see Section 2.2). The remaining categories were developed from a combination of inductive and deductive approaches with the inductive approach given priority [52].

In order to gain information about the frequencies of responses, each of the individual answers were assigned to the inductively formed categories and then counted. Using this method, the quantitative significance of a particular view on culture within the student group became apparent. Repeated responses on the part of a single person were also taken into account during this process. Among others, the different scale levels of culture were quantified due to the fact that some of them were particularly strongly or very rarely represented.

The content-analytical quality criterion of intercoder could be corresponded to as the coding guide was put up for discussion in a group of colleagues.

“The application of the categories to the material” [51] (p. 126) as well as the category constructions could be tested and reviewed that way.

As a method critique, it can be noted that the written questioning of the students often led to quotations being quite short, and the illustration and interpretation of the respective statement were limited as a result.

The analysis of the closed questions in the survey (see Part B in Table 1) was carried out with the help of statistical programs (SPSS and Excel). The primary goal was to identify the frequency values of the individual variables. This descriptive statistical procedure was justified by the nominal or ordinal scale level of the measured values. For this reason, more extensive statistical measures were not used [53]. At these quantitatively closed questions, another criticism can be leveled. Here the formulation has already drawn attention to the existence of nationally specific cultures. There were also no questions on the aspect of transculturality, which might be one reason why the results were highly nationally oriented.

The following presentation of the results aims to answer the main research questions posed at the beginning: (a) which everyday ideas about culture do the students possess? (b) Which spatial references do students produce in the context of culture? Referring to question a, several core concepts were identified, which represent a summarized but not universally valid image of the students’ ideas. In particular, this includes the spatial dimension of culture, which was clear and held the answer to question b.

## 4. Results of the Research

The existing everyday ideas of the participants about culture and space were made up of different core concepts, which are outlined subsequently. All quotations have been translated from German by the authors.

### 4.1. Culture Is Observable

**Figure 1:**
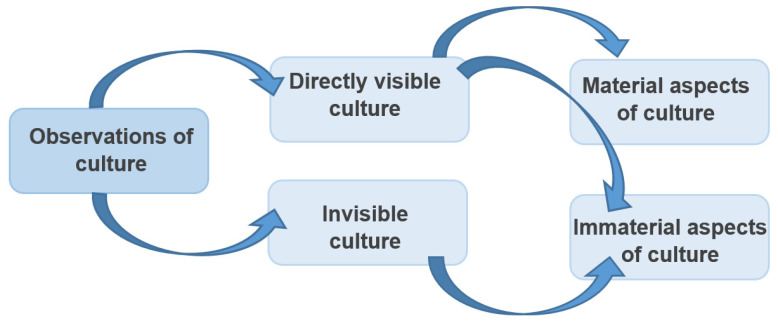
Observations of culture by surveyed students, resulting from open questions, own illustration adapted from Ronja Ege [14] (p. 115).

Culture is observable for all surveyed students, as each respondent mentions at least one of the forms of observing culture listed in Figure 1.

In particular, students perceived directly visible, material aspects that constitute culture for them, such as “Food, clothing” (EV28), or architecture and monuments such as “The [Cologne] Cathedral, World Cultural Heritage” (EV4). In addition, cultures also exist for them in an indirect visible way. Thus, they can be immaterial in nature, such as ways of education, ways of thinking, or major cultural events such as carnivals. For some of the students, directly visible culture was linked to immaterial aspects of culture: “In China, for example, you live in a different culture than in Germany, because there you eat with chopsticks” (EV13). This implies that material forms of culture such as “different food” are representative of a certain national culture, and its representation is manifested in the form of immaterial but visible cultural forms of action (“eating with chopsticks”).

### 4.2. Culture Is Functional

According to the surveyed students, culture in everyday life serves mainly as a means of social assignment and orientation. Based on cultural aspects, demarcations and attributions are made that can maintain individual and collective identities. Thus, culture offers a feasible orientation aid to help order social life on a daily basis. This can also be seen in the following quote: “Culture is good for being different from other cultures and every country is different. But it also connects people if, for example, both have the same or a similar culture” (PT72). Both differences and similarities serve to reproduce homogeneous cultures, which are often mentioned in the context of a national spatial reference. In this respect, the majority of the students possessed a broad understanding of the function of socio-cultural classification and its relevance for the reproduction of community, which is frequently and regularly used in everyday life.

**Figure 2:**
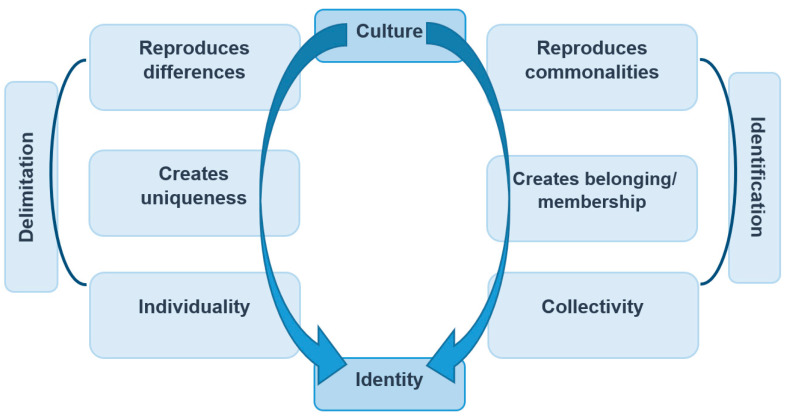
Creation of personal identity through culture among the students, resulting from open questions, own depiction adapted from Ronja Ege [14] (p. 156).

Another important aspect for the respondents, which was apparent from the results of the open survey-questions, was the importance of culture in the generation of identity. The following diagram (Figure 2) schematically depicts the process of creating identity through a culture based on all the category statements relevant to this.

According to the results of the open questions, which were analyzed qualitatively, culture plays a key role in the creation of identity for the pupils. For the respondents, identity is made of two elements: on the one hand, the affiliation of individuals to certain groups is important, where identity is achieved through the creation of cultural commonalities and identification with them; on the other hand, cultural differences create demarcation mechanisms that lead to uniqueness and individual identity, which, for many respondents, includes different ways of living and behaving. Most students understood the independence and autonomy of uniform and unique cultures in comparison to others. To a lesser extent, they referred to the single individual. It was also evident that culture is a fixed part of life for most of the respondents. Moreover, they assume that it is of essential importance for all people. The understanding of this omnipresent significance for the formation of identity is once again made clear by the following quotations: *“Culture is a part of our lives. […] it is just important for everyone”. “Culture keeps me from forgetting who I am” (PT32).*

### 4.3. Culture Is Historically Established and Persists

**Figure 3:**
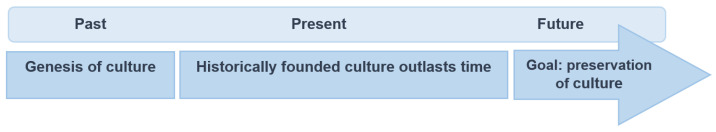
Students’ conception of origin and continuity of culture, resulting from open questions, own depiction adapted from Ronja Ege [14] (p. 121).

The majority of students hold the idea that culture is founded historically and is not influenced by the factor of time. This means that culture is a historical heritage that survives and cannot be changed. The following diagram (Figure 3) represents this idea of the origin and continuity of culture as a synopsis.

For most of the students, the origin of culture lies in the past, which they linked to a specific event occasionally. *“For me, culture began from the time when tools were used for the first time” (EV18).* Accordingly, the existence of human beings seemed to be integral to the emergence of culture and, starting from its source, culture is continuously transmitted, as one student stated: *“[Culture is] something that is passed on over generations. Something that is always the same […] Something children grow up with from an early age” (EV8).* For many respondents, culture consists of historically established ways of living and acting that are constantly reproduced and thus transferred from the past to the present. Consequently, the same culture is considered to exist in the past as well as in the present and future. The persistence of historical roots and traditions is considered to be important by a majority of students and worth preserving. *“Moreover, culture has also been around for a very long time and is therefore a part of history and should be preserved” (PT11).* Occasionally, students referred to influencing factors that undermined and changed this historically developed “own culture.” For example, one student pointed out that some people fear the loss of their solid and enduring basis of identity due to foreign cultures. *Many people are afraid that their culture will be ‘contaminated’ by people from other cultures, this is expressed, for example, in ‘Pegida’ marches […]” (EV12).* PEGIDA is an acronym for “Patriotic Europeans against the Islamisation of the Occident”. The German movement arose out of the great wave of migration in 2015 from the countries of the Middle East, especially Syria. Politically, the movement has a very strong right-wing orientation. The overarching goal is thus to preserve historical culture when confronted with influencing factors, such as current migration.

### 4.4. Culture Has Various Spatial Dimensions

**Figure 4:**
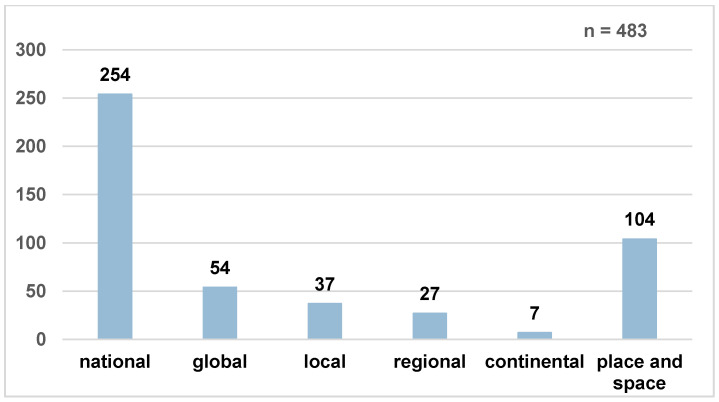
Location of culture in students’ statements according to scale levels, own representation from the results of the open questions, own depiction adapted from Ronja Ege [14] (p. 135).

Space plays a very important role in the students’ concept of culture. The majority of respondents (almost 73%) associate culture with space, as 222 of the total 250 responding students made at least one spatial reference to culture. The results from the open questions regarding the location of culture revealed which spatial distinctions the students make and how often they occur. Figure 4 shows all the geographical scale levels used by the students according to their frequency. Multiple responses were taken into account, the total number of replies was 483.

In the recent disciplinary history of German geography, Ute Wardenga [54] elaborated four spatial concepts that represent a core element of the pluralistic discipline. These concepts include spaces as containers that can be descriptively characterized and delimited from each other on the basis of physical-geographical facts but also anthropogenic aspects. Thus, human-made political borders also provide national spatial demarcations in the sense of a container. Figure 4 shows that a very strong container-based spatial reference [54] was made at the national scale level. Consequently, for the pupils there were nationally-specific cultural aspects that define countries, nations, or states and distinguish them from one another. This result was also reflected in the following quote: *“I think culture is good for distinguishing what nationalities are” (PT113).* Based on an understanding of culture as a way of life and behavior, it seems possible to distinguish people and assign them to nations, as PT113 states. Here, culture explicitly helps to divide people into nationalities. This illustrates the important role of national cultures to the students, which, in addition, is probably also relevant as an identity-forming aspect, as outlined in Section 4.2.

A total of 54 responses referred to a worldwide or international space. In the sense of the scale level ‘global,’ culture is not bound to limited space but is omnipresent for the respondents. *“Culture is everywhere where people live” (EV34).* In this quotation, culture is referred to all human spheres of life thus that, according to the statement, culture and human existence are firmly connected worldwide. Culture could be linked to the local scale level in a total of 37 quotations. The majority of these were spatial references that understand the city as a concrete place to which a respective, locally specific culture is attributed. *“Culture is when you look at an historical city centre, for example” (PT85).* The local culture of a city explicitly includes its urban history, which is materially represented in the form of historical building structure and architecture. In students’ quotations that placed culture in a regional spatial context, the concept of culture is the first and foremost representative of the ways of life and behavior practiced by people in that region: *“The way people […] live in a certain region. This includes food, festivals that are celebrated, the religion, traditions, clothing, etc” (EV38).* Those listed practices of action and life allow the people of the region to be summarized and understood as a homogeneous unit of a regional area. However, statements that referred to culture on a continental scale were only present seven times. This was most likely due to the physical–geographical understanding of the definition of landmass as transnational, political associations such as the EU were not addressed.

In addition to the classical scale levels, students also located culture in *places* and *spaces*, as a total of 104 spatial references did not contain any of the classical scales. Instead, further spatial concepts were used to evaluate and describe the remaining 104 statements (see Figure 4). The English geographical concepts of *space* and *place* understand space as relative, i.e., in dependence on the meaning for persons or groups and their relational structure [55]. In addition to the material aspects of *place* and *space*, the symbolic and emotional meanings also play an important role and contribute to their existence [56]. While *place* defines a particular place through its physical location or its specific socio-cultural meaning and materiality for individuals or collectives [57], *spaces* are understood as space-generating, dynamic networks that exist between people, objects, locations, and their relationships [56]. The majority of the students’ statements could be attributed to these different meanings of places.

For 73 of 104 answers, *places* appeared as specific locations of culture, where buildings and structures are linked to history and practices. *“Culture is in museums, cities, monuments, cemeteries, churches and many other places” (EV37).* Places are understood as symbols for culture and make it visible through their structural existence. It is also possible that the forms of behavior practiced at such places, or certain associated emotions (such as sadness with cemetery), also help to represent that cultural *place*.

In the remaining 31 statements, culture was located as *space*. These spatial references were predominantly expressed through the terms “origin” and “home”: *“In persons, their actions and character, culture often reflects their homeland” (PT82).* PT82 recognized culture through people’s behavior and lifestyles and linked this to their homeland. Accordingly, culture seems to be a link between social practices and the ascribed, non-concrete space of “home(land).” Due to the ambiguity of ‘homeland,’ it remains unclear whether the term (and the cultural practice) referred to a social origin or the country of origin in the sense of a national scale level. The great diversity of the German concept of *Heimat* is only inadequately reflected in this quotation. Different semantic fields of the meaning of the concept of home reveal its great diversity of meaning. Central to this are the territorial-spatial, social, and emotional categories of meaning, which have different connotations in different areas of application [58]. In this respect, the term *homeland* in the quotation can refer both to the place of birth (territorial-spatial category) or to a place where one feels at home through permanent residence (social or emotional category).

It can be critically noted that the spatial concepts presented are handled as generally valid and quantifiable, which is contrary to the basic constructivist idea of the study. However, the students’ positivistically oriented cultural concepts also contain positivistically oriented spatial concepts, which are taken into account here.

### 4.5. Culture Is Non-Transcultural

A total of 13 (5.18%) of the total 250 students directly or indirectly mention approaches to transcultural thinking or transcultural understanding of culture. These references did not necessarily show understanding of culture itself as dynamic but considered the student’s own individual culture as freely selectable by distinguishing between individual cultures due to their different cultural aspects. *“I don’t stick to traditions […] I find my own way a bit…. And mix up some cultural aspects that don’t belong to ‘my’ culture” (PT197).* Here, it becomes clear that the interviewee decides his own cultural practice. The interviewee chooses elements from different cultures and thus composes his own cultural practice. Furthermore, changes and developments seem to take place within nationally limited cultures. *“You can find yourself in a mix of different aspects of different cultures” (PT55).* This change, as well as the composition of an individual culture (PT197), is also understood as dynamic but leaves it as a self-contained, nationally bound entity. A transcultural understanding that recognizes culture as a transnational, hybrid, and in a state of change did not seem to be represented within the surveyed students’ answers.

## 5. Discussion of the Results

The results are a representation of what the German respondents understand by culture and cultural space. In order to both answer the research questions and interpret the results, the most relevant findings are briefly summarized.

(a) Cultures are primarily perceived by the respondents as directly visible. These are both material and immaterial aspects of culture, such as ways of life and behavior. By recognizing and classifying these everyday processes of order become possible. Thus, cultures mainly serve as an orientation aid for the pupils, as similarities within their own culture, as well as differences between cultures, can be established by comparing cultural aspects. This ensures the reproduction of identity by maintaining identification processes with the help of collective belonging based on commonalities. Distinctions and differences with other cultures are also used to maintain the special features and characteristics of one’s own culture. Most respondents considered cultures to be continuous in time. From their point of view, cultural conventions and customs are handed down. This means that culture remains in its “original” state, is static, and cannot be changed. Furthermore, it was shown that space generally plays an important role in the pupils’ conception of culture, as the majority of the student’s related culture to space. In particular, the omnipresence of national cultures stands out, as cultures are located in a container-based space that is based on nation-states. For a small number of students, cultures also exist in other spatial dimensions, but this was not a view that was widespread. A transcultural understanding of culture, as provided by Wolfgang Welsch [17,18,41,42,43], could only be found among the students in a rudimentary way. For them, exchange processes and changes in culture usually consist of a national component. Which everyday ideas about culture do the students possess?

Overall, the students’ ideas about culture and space are largely uniform, as the statements made by students often coincide in terms of content. In particular, three core concepts stood out that strongly contradict the transcultural understanding of culture, according to Wolfgang Welsch [17,18,41,42,43]. These are the notions that culture is historically based, which are maintained and serve as a *heritage* for the preservation of common ancestry or origin, e.g., through the practice of traditions over time. The national positioning of cultural aspects, which reproduce a specific national-spatial collective identity, contributes significantly to this. Similarly, cultural characteristics serve to reproduce collective belonging and to maintain a collective identity. This is especially true for the affiliation to national cultures, which is strongly represented among the students. All aspects reflect an essentialist understanding of culture and are contrary to the idea of making culture, according to Wolfgang Welsch [17,42,43].

(b) Which spatial references do students produce in the context of culture?

As mentioned before, the spatial reference of culture, especially in the context of national–spatial, is very important in the surveyed students’ imaginations. As the ubiquity of national culture in everyday life suggests, cultures are primarily located through a container-based spatial reference based on nation-states. Furthermore, for the students, cultures also exist in specific places that symbolically represent cultures (buildings) but are also places with a certain type of social interaction (cemeteries). According to the surveyed students, places as symbolic locations of cultures can be material or immaterial, clearly visible or hidden. In this respect, places reflect the cultural practice of a specific place within a container and thus serve the representation of and identification with the everyday cultural practice of life. Accordingly, local as well as national space are essential dimensions that students use to explain the functionality of culture. Based on the main results of this study, it can be assumed that the students have a predominantly holistic–essentialist understanding of cultural space. The concept of the modern nation, which is of such importance to the students, and the accompanying cultural classification and orientation, is historically still young [59]. Nevertheless, the nation represents the essential core of identity reproduction for collectives [60]. Through social characteristics, the nation is objectified into a specific cultural nation and understood as universally valid. Historically, too, collective identities originate from a common ancestry in a long-established territory. At that time, many ethnic groups had a common settlement area, and within this area, the development of uniform characteristics for the constitution of the community could take place [59]. Thus, the spatial origin is already anchored in pre-modern times as a relevant aspect for the reproduction of a we-consciousness and is still a substantial element in the maintenance of collective identities.

These long-established, identity-forming social structures are being challenged by social change and globalization influences. Both the displacement of national–cultural identities and the formation of a fundamentalist return to the local as an antithesis to globalization is predicted [1]. Since the students have a strongly nationalistic understanding of culture and space, it can be assumed that national–cultural collectives will not be undermined by globalization. Rather, it seems that the preservation of spatially bound national cultures provides security in times of unpredictable changes [1]. Most likely, the students are confronted in their everyday lives with nationalistically oriented and stereotypical concepts of culture that remain unquestioned and are taken for granted. Thus, in the current media discourse, historically based, geographical world views are reproduced that are orientated towards natural determinism, biologism, and regional studies, such as the recurring example of migration as a danger in the political context. Donald Trump’s plan to build a border wall to protect the nation from undesired migrants, or Victor Orbán’s terse statement that all migrants are terrorists [61] shows the representation and reproduction of supposed danger and insecurity through migration movements. These concepts can be found in current geographical world views, such as the clash of civilisations by Samuel Huntington [62], as well as in many travel reports and travelogues, which contribute significantly to the formation of an overall social worldview [22]. In geography textbooks, too, contents such as Albert Kolb’s [10] and Jürgen Newig’s [11] concept of major civilizations, which can be spatially located, contribute to cultures being understood as classifiable containers of a specific space [5,8]. The students’ already existing thinking in cultural dichotomies should not be additionally reinforced with textbook content of this kind [5] in order to avoid dangers such as xenophobia. Instead, the learning conditions should be used as a way in which to enable the students to change their perspective on the topic, examining culture and space as a human-made concept in a constructivist sense.

## 6. Conclusions and Outlook

It does not come as a surprise that the students’ ideas about culture and space are strongly holistic–essentialist in orientation and rarely show any significant transcultural aspects. In order to counteract this outdated way of thinking, which fosters dangerous concepts such as racism, it makes sense to incorporate the concept of transculturality within geography lessons. Although students regularly encounter and actively reproduce transcultural phenomena in their everyday lives, it is clear that they are not aware of them in any way. A suitable example of such a phenomenon is the sub- and pop culture of hip-hop, which is very popular among the majority of adolescents and young adults. This could be confirmed in our own study with 100 pupils [63]. The intrinsic motivation to deal with the topic in the classroom is thus given as a profitable basic prerequisite. In addition, hip-hop can be considered a prime example of transcultural phenomena due to its worldwide expansion, as well as its local-specific content and productions. Through the interlocking of globalization and local specificity, hip-hop has a global spatial reference and accordingly is also a global phenomenon [64]. This aspect is of importance for a profound understanding of transculturality. More specifically, a lesson could be developed in which students are introduced to the transcultural phenomenon and gain knowledge about its expansion, global spatial reference, and reproduction mechanisms. Further transculturally oriented lessons should be developed and tested. For example, the topic of globalization of food could be taken up according to the slogan “You are what you eat.” By analyzing their daily consumption, the students can uncover their own glocal food culture. The public space can also be exposed as transcultural by analyzing the culinary offer in a city [65]. In addition to geography, other subjects could also be included to exploit the breadth of the subject matter [66]. Furthermore, it could be analyzed to what extent the essentialistic everyday ideas were modified by this approach.

## Figures and Tables

**Table 1:** Investigation procedure adapted from Ronja Ege [14] (p. 86).

Overview of Investigation Setup			
*Sub-Surveys*	*Interview Questions*	*Investigation Interest*	*Analysis*
**1. Explorative Pre-Study** Written survey with a qualitative open question. Sample size: 53 students	Qualitative open		
	“What, how and where is culture for you?”	Mapping the spectrum of the thematic field of students’ ideas about culture and space.	Content analysis according to Mayring [50,51]. The results helped with the orientation and development of the core survey.
**2. Core Survey** written standardized survey with a qualitative open and quantitative closed questions. Sample size: 197 students	Part A: Qualitative open		
	“Just off the top of your head, what is culture for you?”	Insight into the students’ symbolism of culture through description of an abstract concept.	Content-analytical analysis, which takes both content-structuring and ordinal scaling procedures into account. Implementation of qualitative and quantitative analysis steps [51]. Combination of inductive-deductive approach to create a category system [52]. Further generalization of the results through occupation frequencies [51].
	“What is culture good for?”	Evaluation/assessment of culture and estimation of its relevance.	
	“Where is culture expressed for you?”	Insight into different connections between culture and space and the creation of spatial relationships.	
	Part B: Quantitative closed questions to tick in a Likert scale. Checkbox options: exactly true, approximately true, somewhat true. not true at all, don’t know.		
	“Culture as a way of life (such as language, food, traditions…) varies from country to country.”	Quantitative assessment of the national spatial reference.	Descriptive statistical procedure to identify the frequency manifestations of the individual variables, respectively statements.
	“I feel I belong to a particular culture.”	Quantitative assessment of personal connection with the subject matter.	
	“Culture is important because it connects people.”	Quantitative assessment of the importance and value of culture as an organizing tool.	

**Table 2:** Excerpt from the category system giving coding examples, adapted from Ronja Ege [14] (pp. 101–104).

Extract from Category System			
*Category*	*Subcategory*	*Explanation*	*Text Examples*
**Observation of culture**	Visible observations of culture Invisible/indirectly perceptible observations of culture	Perceptible attributes of culture that are observable Values, norms, or ideals that are attributed to moral, social-societal action that cannot be observed directly.	*“Mosques, synagogues, churches” (PT128).* *“[…] in another sense it can mean to me that one has values to which one holds on to, or even rules that everyone follows” (EV19).*
**Functions of culture**	Expression of differences or commonalities Culture is the own or the foreign	Subsumption of culture as functional application of everyday life as well as social Classification of people and thus orientation.	*“Culture is good for being different from other cultures and that every country is different. But it also connects people if, for example, both have the same or a similar culture” (PT72).*
**Genesis of culture**		Aspects that contribute to the emergence, genesis, or development of culture.	*“Cultures are often something very old that has been around as long as people have been living together” (PT44).*
**Placing and locating culture**	Cultural space by scale levels (global, continental, national, regional, local) Space and place according to a constructivist understanding	Inductively collected and summarized aspects of space and place in the context of culture. Adding deductive considerations of spaces, places, and scales from a scientific point of view and linking with inductively collected aspects out of texts.	*“[Culture is when] you look at an ancient part of the city, for example” (PT85).* *“In persons, their actions, and their character, culture is often reflected in their homeland [sic]” (PT82).*
**Transcultural approaches**		Statements that included transcultural or glocal aspects of culture.	*“I don’t stick to traditions […] I somewhat find my own way… And mix some cultural aspects which do not belong to ‘my’ culture” (PT197).*

## Data Availability

The data presented in this study are available on request from the corresponding author. Due to the protection of privacy all data were made anonymous.
